# Standards for reporting interventions in clinical trials of cupping (STRICTOC): extending the CONSORT statement

**DOI:** 10.1186/s13020-020-0293-2

**Published:** 2020-01-31

**Authors:** Xuan Zhang, Ran Tian, Wai Ching Lam, Yuting Duan, Fan Liu, Chen Zhao, Taixiang Wu, Hongcai Shang, Xudong Tang, Aiping Lyu, Zhaoxiang Bian

**Affiliations:** 1 Chinese Clinical Trial Registry (Hong Kong), Hong Kong Chinese Medicine Clinical Study Centre, School of Chinese Medicine, Hong Kong Baptist University, Hong Kong, Hong Kong SAR, China; 2 Institute of Basic Research in Clinical Medicine, China Academy of Chinese Medical Sciences, Beijing, China; 3 Chinese Cochrane Centre, West China Hospital, Sichuan University, China Trial Registration Center, Chengdu, Sichuan China; 4 Key Laboratory of Chinese Internal Medicine of Ministry of Education and Beijing Dongzhimen Hospital, Beijing University of Chinese Medicine, Beijing, China; 5 Xiyuan Hospital, China Academy of Chinese Medical Sciences, Beijing, China

**Keywords:** Cupping, CONSORT extension, Randomized controlled trial, Reporting guideline

## Abstract

**Background:**

The standards for reporting interventions in clinical trials of cupping (STRICTOC), in the form of a checklist and explanations for users, were designed to improve reporting of cupping trials, particularly the interventions, and thereby facilitating their interpretation and replication.

**Methods:**

A group of clinical experts, methodologists, epidemiologists, and editors has developed this STRICTOC checklist through a comprehensive process, including registration of this guideline, literature review, solicitation of comments, consensus meeting, revision, and finalization.

**Results:**

The STRICTOC checklist includes 6 items and 16 sub-items, namely cupping rationale, details of cupping, treatment regimen, other components of treatment, treatment provider background, and control or comparator interventions. Illustrative examples of each item are also provided.

**Conclusions:**

It is intended that the STRICTOC, in conjunction with both the main Consolidated Standards of Reporting Trials (CONSORT) Statement and extension for nonpharmacologic treatment, will raise the reporting quality of clinical trials of cupping.

*Trial registration* We have registered this study on the Enhancing the Quality and Transparency of Health Research (EQUATOR) Network: http://www.equator-network.org/library/reporting-guidelines-under-development/reporting-guidelines-under-development-for-clinical-trials/#STRICTOC.

## Background

Cupping is an ancient medical technique in which a small area of local suction is created on the skin [1]. The earliest record of cupping is in the Bo Shu (an ancient book written on silk), which was discovered in an ancient tomb of the Han Dynasty in 1973 [2]. Cupping practice has been, and continues to be, a vital part of nearly every traditional medical system in Asia (e.g., China, Korea, the Middle East) and Europe [3–5].

Recently, there has been a renewal of popular interest in the use of cupping because some athletic stars have received cupping for musculoskeletal care [6]. As more people seek cupping to deal with their health problems, more doctors are researching the underlying mechanism(s) and clinical applications; there is evidence that cupping can reduce some types of pain [7–10]. Notably, the number of clinical trials of cupping has increased [11, 12]. However, some scholars have indicated that the quality of cupping trials is generally poor, especially in the assessment of the risk of bias for important outcomes within each trial [13]. Incomplete reporting is a significant reason for this phenomenon and results in a great deal of avoidable waste in research [14]. In particular, the inadequate reporting not only compromises the value of the cupping treatment but also may affect reviewers’ and readers’ judgments about the efficacy and safety of cupping in general, inviting skepticism and criticism [15].

The Consolidated Standards of Reporting Trials (CONSORT) Statement [16] and its extensions have substantially improved the reporting quality of randomized controlled trials (RCTs) [17, 18]. CONSORT extensions for pragmatic trials [19], nonpharmacologic treatments [20], herbal interventions [21], Chinese herbal medicine formulas [22], acupuncture interventions [23] and moxibustion interventions [24] have been developed, but none of these can be used for reporting clinical trials of cupping because specific aspects of the interventions (e.g., the rationale of cupping selected, devices and techniques of cupping therapy, description of sham cupping, etc.) are not covered by available guidelines. Therefore, it is necessary to develop standards for reporting interventions in clinical trials of cupping (STRICTOC) congruent with CONSORT and its extensions.

## Methods

The STRICTOC is developed based on the CONSORT Statement. The framework of the revised standards for reporting interventions in clinical trials of acupuncture (STRICTA) [23] and the standards for reporting interventions in clinical trials of moxibustion (STRICTOM) [24] was referred. Development proceeded in six specific steps: Firstly, the STRICTOC working group (Additional file 1: Appendix S1) was formed, and registered STRICTOC on the EQUATOR Network based on the need for extending the CONSORT [25]. Secondly, authors (XZ and RT) thoroughly reviewed the clinical trials on cupping and flagged items on the CONSORT, STRICTA and STRICTOM checklists that needed extension or modification according to the specificities of cupping. Thirdly, the working group members reviewed the suggested checklist and provided comments. Fourthly, authors (XZ and ZB) used the comments from the STRICTOC working group to draft a list of items that needed to be included in the STRICTOC checklist. Fifthly, a face-to-face consensus meeting comprised of 11 professionals (Additional file 1: Appendix S2) in clinical practice, trial methodology, epidemiology, statistics, and medical journal editors, was held in Lanzhou, China, on 19th July 2017. More than half of the attendees were Chinese medicine (CM) professionals who used cupping in their practice. During the meeting, the experts revised and finalized each item of the checklist. Finally, after the meeting, working group members edited the STRICTOC checklist, identified one or more examples of good reporting for each item, and drafted the manuscript. The STRICTOC working group members presented the rationale of development of STRICTOC among different academic conferences to solicit the comments [26–28], and finalized the manuscript in October 2019.

## Results

The STRICTOC checklist include six items and sixteen sub-items (Table 1). The explanations and examples of each item are provided below. There was agreement that the STRICTOC checklist could be applicable for reporting a broad range of clinical studies with cupping intervention, not limited to the RCTs (e.g., uncontrolled outcome studies, case reports, etc.).

**Table 1:** Checklist of items for reporting trials of cupping

No.	Item	Detail
1	Cupping rationale	1a. Style of cupping (e.g., Chinese medicine, dry cupping, wet cupping, etc.)
		1b. Reasoning for cupping provided, based on historical context, literature sources, and/or consensus methods, with references where appropriate
		1c. Whether the cupping treatment is individualized or not
2	Details of cupping	2a. Patient posture during the cupping
		2b. Devices used for cupping, such as type of cupping set, size, manufacturer, and material (e.g., herbal, needle, moxa, water) inside the cup, if any
		2c. Name and number of acupoints/meridians/locations (if no official name) used for cupping
		2d. Number of cupping units and/or cupping time per location (mean or range where relevant)
		2e. Procedure and technique for cupping (e.g., weak/light cupping, medium cupping, strong cupping, moving cupping, light-moving cupping, needle cupping, hot needle and moxa cupping, empty/flash cupping, bleeding/wet/full cupping, herbal cupping, water cupping and ice cupping)
		2f. Responses sought from participants (e.g., warm feeling, skin reddening, ring mark, etc.)
		2g. Precautionary measures to adverse events (e.g., skin blister, scald, or bleeding), and management, if any
3	Treatment regimen	Number, frequency and duration of the cupping sessions
4	Other components of treatment	4a. Details of other interventions administered to the cupping group (e.g., acupuncture, moxibustion, massage, herbs, exercises, lifestyle advice)
		4b. Setting and instruction of treatment to the cupping providers and the participants
5	Treatment provider background	Description of treatment provider(s) (qualification or professional affiliation, years in cupping practice, and other relevant experience for professional)
6	Control or comparator of cupping	6a. Rationale for the choice of control or comparator of cupping
		6b. Precise description of the control or comparator. If another form of cupping or cupping-like control is used, provide details as for Items 1 to 3 above

This STRICTOC checklist, which should be read in conjunction with the explanations of each item provided in the main text, is designed to extend CONSORT 2010’s item 5 when reporting a cupping trial

### Item 1: cupping rationale

#### Item 1a

Style of cupping (e.g., Chinese medicine, dry cupping, wet cupping, etc.).

##### Explanation

Cupping has a long history in many cultures and is characterised by a broad diversity of styles and approaches in Asia and the West [29]. There are many styles of cupping therapy, differing by geographical region, culture, and practitioners’ experience and skills. For example, in Chinese medicine, there are various cupping styles including flash cupping, retained cupping, sliding cupping, medicated cupping, cupping with needle retention, bloodletting puncture with cupping and hydro-cupping [4]. In the Middle East and Europe, dry and wet cupping are the main types; however, Arabian scholars have categorized cupping into specific styles-based on various technical (e.g., massage cupping), power of suction (e.g., pulsatile cupping), method of suction (e.g., automatic suction cupping), area treated (e.g., abdominal cupping) or purpose (e.g., sports cupping) [30]. Thus, authors should state the overall style or approach of the treatment, in order to help readers contextualize the trial within the range of current clinical practices. If the selected style of cupping is entirely novel, either in terms of device or technique, we recommend authors to provide a brief description for it.

##### Examples

Note: For the following and for all subsequent examples, the references reported in the original published studies are not provided here for reasons of brevity.

i. The aim of our study was to investigate the effectiveness of dry pulsatile cupping in reducing pain and improving back function and quality of life in patients with nonspecific chronic low back pain (cLBP). We therefore designed a three-armed, randomized controlled trial comparing (i) strong negative pressure pulsatile cupping plus paracetamol on demand (pulsatile cupping) vs. paracetamol on demand only (control, no cupping), and (ii) weak negative pressure cupping plus paracetamol on demand (minimal cupping) vs. paracetamol on demand only (control, no cupping) [31].
ii. Fifty-seven patients with nerve-root type cervical spondylosis (NT-CS) were recruited from the Affiliated Health Care Hospital, Tianjin University of Traditional Chinese Medicine from October 7, 2012 to May 14, 2013 and randomly divided into the Wet cupping therapy and Jiaji acupoint-acupuncture groups according to random number table [32].
iii. The cupping group received fire cupping therapy at three acupuncture points, SI 15, GB 21, and LI 15 [33].

#### Item 1b

Reasoning for cupping provided, based on historical context, literature sources, and/or consensus methods, with references where appropriate.

##### Explanation

The authors should provide the underlying rationale for the chosen cupping from the perspective of historical context, literature sources, and/or consensus methods. For cupping based on traditional practice, the historical or cultural background should be provided. If the style of cupping belongs to CM, the relevant theory should be described, including the diagnosis according to CM pattern/syndrome (e.g., excess or deficiency pattern), the rationale for point selection (e.g., Ashi point or meridian acupoint), and mechanism of cupping method (e.g., promoting circulation of qi and blood) [34]. If the cupping selection comes from a consensus, full details of how the consensus was reached should be reported (e.g., expert clinical panels and/or practitioner surveys). Literature references could be the basis of the cupping selection; and, if so, it is essential to provide the relevant citations, such as previously published clinical evidence(s), biomedical experiment findings, pilot studies or systematic reviews, if applicable. In addition, any evidence of the benefits and/or harms of the cupping studied, and any cupping-prohibited situation should be reported.

Authors are encouraged to cite the published works that are readily available. If a reference is not publicly available, detailed information could be provided in the manuscript or supplementary materials. For fully individualized trials, as practitioners normally do, it is appropriate to specify the qualification of practitioners and provide a brief description of how they conduct the cupping treatment. For most clinical trials of cupping, the intended interventional procedures are defined in advance, but there could have difference when actually applied during the trial. If so, the details of the actual procedure should be provided in the final version of the publication.

##### Examples

i. In Korean medicine (KM), cupping has been employed for facilitating the circulation of qi and blood and removing blood stasis using cupping therapy devices through suction and negative pressure. According to a recent survey of Korean medicinal doctors, cupping is primarily used for the treatment of musculoskeletal diseases (96%), and the most frequently used points for cupping are the neck and shoulder (94%) [35].
ii. Cupping therapy is a 2000-year-old form of complementary and alternative medicine (CAM), and depending on its application is classified as dry or wet cupping. Wet cupping involves bloodletting, that is, the evacuation of morbid humor from affected areas. Dry cupping involves diverting morbid matters from one site to another by applying quick, vigorous, rhythmical strokes on intact skin (without bloodletting). Therefore, dry cupping is considered to be a noninvasive and inexpensive technique. More specifically, in this technique, the underlying tissues are pulled into the suctioning cupping glass by heat production to increase the local blood and lymphatic circulation. Although this technique has been used in the treatment of numerous conditions including excessive menstrual bleeding, edema, scrotal hernia, sciatica, hydrocele, postpartum perineal pain, chronic neck pain, and low back pain, we are not aware of any previous studies testing the effectiveness of dry cupping in the treatment of postoperative nausea and vomiting (PONV) [36].
iii. There are mainly two methods of performing wet cupping. The first one is mainly used in Chinese Medicine and also in some German studies…… Taiba Theory is the latest theory explaining the therapeutic effect of wet cupping or hijama…… There are several systematic reviews that have been done to identify the efficacy of wet cupping on certain conditions…… A relevant systematic review was implemented which including all the clinical trials that studied the efficacy of cupping on hypertension…… An important randomized clinical trial (RCT), done by Zarei et al. assessing the efficacy of wet cupping in the treatment of hypertension was conducted in Iran and published in 2012…… The objective of this study was to evaluate the efficacy of wet cupping therapy (Hijama) on the management of high blood pressure among hypertension patients [37].

#### Item 1c

Whether the cupping treatment is individualized or not.

##### Explanation

Whether the cupping treatment is individualized for different participants by treatment providers should be described. Generally, the treatment providers are required to apply a standardized procedure of cupping to each participant to minimize variation across the trial treatments. However, many styles of cupping, whether based on traditional theories or Westernized concepts, are individualized in routine practice [38]. Specifically, variations may include the selection of point or treatment area, the power of suction, types and sizes of cups, materials inside cups, or treatment protocol based on different individual’s constitutions. Thus, if a trial is designed to apply, partially or fully, individualized treatment, it should report the possible variations. Additionally, the underlying rationale of such study design should be reported.

##### Examples

i. The selection of points and cupping methods were decided by practitioners after a brief consultation with the participants at each treatment session and was based on the basic guidelines of treatment from acupuncture textbooks and previous studies [35].
ii. In the dry cupping therapy group, a plastic cupping bell (Kangzhu 6-Cup Biomagnetic Chinese Cupping Therapy Set, Model B1 × 6, Kangzhu, Beijing, China) was applied to the painful site for 10 min in each session. A manual hand pump was used to create the vacuum for suction. The intensity of the vacuum was based on subject tolerance. In the electrical stimulation therapy group…… The electrodes were placed around the painful site, and pre-modulated interferential current electrical stimulation was conducted for 10 min. The intensity of the current was increased to patient tolerance at the sensory level. The carrier frequencies were 4000 Hz and 4000–4150 Hz. The beat frequency was 80–150 Hz [39].
iii. Participants who were assigned to the treatment group received wet-cupping therapy 3 times per week for 2 weeks…… Treatment points were located bilaterally at BL23, BL24 and BL25 according to the WHO Guideline for Acupuncture Point Locations. Each time, the practitioners chose 2 points that were the most painful sites of the points when pressed and palpated manually. We chose BL23, BL24 and BL25 for treatment points because these points are located in the low back and are the painful points where back pain patients usually feel discomfort. These points are also frequently used in traditional Korean medicine to treat persistent non-specific low back pain. In the case where there were no painful points, we chose the bilateral BL25 [40].

### Item 2: details of cupping

#### Item 2a

Patient posture during the cupping.

##### Explanation

During the cupping treatment, the posture of a patient should be comfortable, natural, and well fit the needs of the treated location. To standardize the treatments, details of posture could be a factor for ensuring the repeatability of trials. Thus, authors should report the specific posture(s) used for different treatment areas, such as prone, sitting, or lying sideways. If both front and back points are involved in the treatment, the posture of the patient of both sides should be described.

##### Examples

i. Patients lay prone on a massage couch with their upper torso bared [41].
ii. The patient took a sitting position or lying position according to the affected site fully exploring the positions for bloodletting [42].
iii. The skin on the subject’s back was exposed with the subject in the prone position [32].

#### Item 2b

Devices used for cupping, such as type of cupping set, size, manufacturer, and material (e.g., herbal, needle, moxa, water) inside the cup, if any.

##### Explanation

Details about the specific types of cupping devices used, including the materials (e.g., plastic, glass, rubber, bamboo, ceramic, metal, and silicone), size (e.g., mean or range of diameter where relevant), product name and manufacturer, if applicable, can help reader to understand the trial and repeat the treatment, if needed. For trials using a variety of different cupping sets, it is necessary to report the details of each type. For materials inside cups, they commonly include needle, moxa, herbs, or water while some new cupping devices contain magnets, a laser probe, or electrical stimulant.

##### Examples

i. Four to eight 50–100 mm diameter acrylic glass cups were placed on the skin, and air was partially evacuated from the cups by means of a mechanical device (Pneumatron^®^ 200S, Pneumed GmbH, Idar-Oberstein, Germany) [43].
ii. For the cupping therapy, various sizes of sterile disposable cups (Seongho Trade & Company, Korea) from 1.5 to 5 cm in diameter were used…… For wet cupping, the skin was punctured 6 times to a 2-mm depth around the cupping sites with 26-gauge disposable lancets (Tianjin Haing Lim Sou Won Medical Instrument Co., China) [35].
iii. The vacuum in the cupping glass was created with a cup glass (4–7 cm diameter) made of plastic with non-return valve and a vacuum gun [44].

#### Item 2c

Name and number of acupoints/meridians/locations (if no official name) used for cupping.

##### Explanation

The name and number of acupoints/meridians/locations (if no official name) should be reported as accurately as possible. Standard nomenclature should be used for acupoints (e.g., LR 10) and/or meridians (e.g., conception vessel), while anatomical location can be used for the specific area where there is no standard name. For most CM practitioners, cupping is often performed on meridians and acupoints based on the CM theory of channels and collaterals [45, 46]. For non-CM trained practitioners, some scholars have devised a “four-zone cupping therapy map”, comprising the upper zone, the middle zone, the lower zone, and the sacral and lower limb zone. Each zone has an accurate description of range, such as “The Lower Zone starts from the 11th thoracic vertebra (T11) and terminates at the fourth lumbar vertebra (L4)” [47]. Methods used to identify the location(s) treated in clinical trials should be reported with a detailed description or citing references. Such information is necessary for readers to replicate the study, reduce ambiguity and enhance transparency. Also, whether the cuppings are applied unilaterally or bilaterally should be described.

In addition, the total number of treated points with cupping per subject per session should be provided, or the mean/range of points’ numbers selected across all participants should be reported. When the individualized point prescriptions are applied in the clinical trials, authors should consider the best way to present the information adequately. For example, for partial individualized cupping treatments, the essential formula should be provided, as well as all modified points or the most commonly used modified points (with percentages) according to different syndromes should be listed. For fully individualized prescriptions, all the treated points should be provided; alternatively, if the list is extensive, only the most frequently used points should be reported.

##### Examples

i. Hijama (wet-cupping) was performed at four sites (Figure 1). The first site was between the two scapulae, opposite the T1–T3 scapular spine. This is the recommended site for treatment of hypertension in an RCT previously done in Iran. This area is called Al-Kahil in Arabic. The second site was located on the seventh cervical vertebra. This site was used in the uncontrolled observational study performed in China on the efficacy of wet-cupping for hypertension, and it is called GV14 in Chinese medicine. The other two sites were on both sides of the neck. They are located two fingers posterior to the angle of the mandible on both sides, just below the skull bone, on the hairline. These two areas are called Al-Akhdaain in Arabic, and they were added because they are recommended areas in Islamic literature for general healing along with Al-Kahil [48].
ii. In each session, two out of six treatment points were selected from the bilateral bladder meridian (BL): BL23, BL24 and BL25 [49].
iii. The cupping glass was slowly conducted parallel to the spine caudally in the course of the bladder meridian and after reaching the lower lumbar spine direction cranially to the lower part of the cervical spine…… The same procedure then followed for the contra lateral back side [44].

#### Item 2d

Number of cupping units and/or cupping time per location (mean or range where relevant).

##### Explanation

The total number of cups used per subject per session should be reported. For individualized treatments, it is necessary to report the ‘‘mean ± standard deviation’, or ‘median and range’ of numbers across all participants. Among different regions, cultures, and medical systems, the number of cups used varies. For example, Western practitioners often use between 5 and 12 cups during one session. In the Far East, however, it is normal to see up to 60 cups being used during one session [50]. For clinical trials, however, it is essential to design an appropriate number during one session, and supporting references, underlying rationales, and/or any particular arrangement during the cupping procedure should be clearly described. The duration time of cupping per point in one session should also be reported as either a ‘mean ± standard deviation’ or ‘median and range’. Researchers should clearly report the cupping time per point, between the production of suction on the skin and removal of cups (retention time). If the flash/empty cupping (without retention time) used, the frequency and/or repeated times (mean or range) during a fixed period per point per session should be provided.

##### Examples

i. Three glass cups with a size of 6 cm (external diameter) was applied below and above each breast with an interval of half an hour for 10 min [51].
ii. Participants randomized to the minimal cupping group received 8 cupping sessions (each 8 min) in 4 weeks with a HeVaTech PST 30 pulsatile cupping device, also with two silicone cups and a weaker negative pressure around − 70 mbar and suction intervals of 2 s [31].
iii. Four to eight 50–100 mm diameter acrylic glass cups were placed on the skin, and air was partially evacuated from the cups by means of a mechanical device (Pneumatron^®^ 200S, Pneumed GmbH, Idar-Oberstein, Germany)…… After 10 to 15 min cups were removed…… One treatment session lasted about 30 min in total [43].

#### Item 2e

Procedure and technique for cupping (e.g., weak/light cupping, medium cupping, strong cupping, moving cupping, light-moving cupping, needle cupping, hot needle and moxa cupping, empty/flash cupping, bleeding/wet/full cupping, herbal cupping, water cupping and ice cupping).

##### Explanation

The procedure and technique for delivering cupping are essential for replication, it is needed to report with sufficient details. The selection of different procedures and techniques of cupping is always determined according to the condition of patients and treatment areas [52]. For example, with fire cupping, the bigger the fire, the stronger the vacuum, the greater is the suction. If possible, the pressure (mean or range) inside the cup is provided for readers to understand the strength (e.g., light, medium, strong) of the cupping. If special techniques are used, for example, needle cupping (applying the cups over acupuncture needles), hot needle and moxa cupping (placing the cups over needles or burning moxa), wet cupping (applying the cups to locations after bloodletting therapy), herbal cupping (applying the cups over an herbal prescription on the skin), as many precise details of needling, moxa, herbs, bleeding monitor and amount should be described. If applicable, relevant schematic diagram or video could be provided to explain how to implement the treatment.

##### Examples

i. The cupping procedure was performed as follows: The patient laid down on the massage bed with her upper torso bared. Three glass cups with a size of 6 cm (external diameter) was applied below and above each breast with an interval of half an hour for 10 min. The vacuum was created with 3 double-walled glass cups, with a size of 6 cm (external diameter) and were held inverted over an open flame to heat the air inside, after which the glass cups were placed below and above each breast at an interval of half an hour for 10 min (one up and two down). As the air inside the cups cooled, vacuums were created, drawing up the skin within each cup [51].
ii. The cupping procedure is as follows: (1) an alcohol swab is ignited, (2) the burning swab is quickly placed inside the cup and withdrawn, (3) the cups are placed over the three acupuncture points, (4) the cups were then removed after 10 min, and (5) the same process was repeated for the same amount of time on the subject’s left side (Figure 2(a)) [33].
iii. The cupping technique involved the static application of high-quality polycarbonate cupping jars (meridian cupping set, Durham-USA) on the three tTrPs for 5 min. Negative pressure inside cups was produced through a rubber pump which sucked the air out of the cups (Figure 6) [53].
iv. Participants randomized to the pulsatile cupping group received 8 cupping sessions (each 8 min) in 4 weeks with a HeVaTech PST 30 pulsatile cupping device and a negative pressure between − 150 to − 350 mbar) and suction intervals of 2 s (see Fig. 1) [31].

#### Item 2f

Responses sought from participants (e.g., warm feeling, skin reddening, cupping mark, etc.).

##### Explanation

As a kind of external treating method, the effect of cupping therapy is not only depending on the levels of practitioners’ techniques, but also relating to the acquired sensations from patients. To standardize the reporting of cupping details, we recommend authors to provide information about participants’ responses to the treatment. For example, in response to general cupping patients should experience a warm, pulling or stretching sensation on the skin, but not pain. If the treatment expected special sensations, please specify. Besides, cupping usually leave marks on the skin; the intensity of these marks depends on the length of treatment time and the strength of the suction achieved [54]. Thus, a description of cupping marks on participants should be provided. If sham cupping control is included in the trial design, it is necessary to report the information of sensations and responses sought from participants both in experiment and control groups.

##### Examples

i. Moving cupping up and down was applied in the following way: with couplant smeared on the back, cupping after flash of fire was made along bilateral side of the spinal column by evenly moving cupping up and down to cause appearance of atropurpureus dots [55].
ii. The glasses were removed after 10 to 20 min depending on the colour of the circular so-called cupping marks, which range from slightly rose to dark pink. Cupping marks usually fade away completely after 2–4 days [41].
iii. Patients in both cupping groups were told that they might feel suction initially, but this sensation would usually disappear after a few seconds, because receptors in the skin would adapt to the stretch of the skin [43].

#### Item 2g

Precautionary measures to adverse events (e.g., skin blister, scald, or bleeding), and management, if any.

##### Explanation

As cupping therapy is directly delivered on the naked skin, it could involve some risk of adverse events such as burning, blistering and bleeding depending on the duration of treatment time, strength of suction, and constitution of the patient receiving the cupping [56]. These adverse events directly affect the compliance of patients and the rate of drop-out [57]. Therefore, any precautionary measures for the possible adverse events should be reported. For cupping trials, it is good practice to sufficiently explain the benefits and the potential adverse effects of cupping therapy to participants before treatment [58].

If some harms or adverse events happened, detailed information should be reported according to the Item 19 Harms of the CONSORT checklist [16]. If applicable, special management during and/or after cupping should be reported. For example, cupping mark is inevitable in the treatment, sometimes strong cupping, if performed for a long time, can cause blisters inside the ring mark. In these cases, management details should be reported, such as “a sharp, sterilized instrument (e.g., acupuncture needle) is used to burst the blister and drain the fluid out, and then the sterilized gauze is used to cover the area and keep it dry for few days” [59]. For wet cupping, a small amount of blood (e.g., a few drops) is usually sucked into the cup and later removed with a cotton ball [60]. If unintended bleeding happened in the trial, the special treatment and the reason (e.g., inappropriate operation by a treatment provider or the sensitive constitution of an individual patient) of this harm should be reported.

##### Examples

i. Mechanical suction was preferred in this study to avoid burning the skin [61].
ii. For female patients, the suction should be moderate to avoid injuries of the tissues of the mammary gland [62].
i. At first, light to medium suction was applied. Depending on tolerability, the suction was gradually increased during therapy [44].
ii. In addition, to avoid possible adverse events related to the wet-cupping procedure, practitioners treated participants according to the pre-defined clean wet-cupping technique procedure (Table 1) [40].

#### Item 3: treatment regimen

Number, frequency and duration of the cupping sessions.

##### Explanation

The planned number of sessions, frequency and duration of each cupping session should be clearly documented in “Methods” section, while the actual number of cupping sessions received by participants should be reported in “Results” section. If there are variations among the participants, the mean or the range of number, frequency, and duration of treatment sessions between all participants should be reported.

##### Examples

i. This study therefore aimed to test the efficacy of 12 weeks of a partner-delivered home-based cupping massage…… Two treatment sessions per week of 10–15 min’ duration each at comfortable intensity were recommended [63].
ii. In the present study, three hijama sessions were conducted, once per month for 3 consecutive months. In Islamic literature, it is recommended to perform himaja on the 17th, 19th and 21st days of the Arabic calendar months (lunar month). In this study we restricted from carrying out the hijama sessions on those specific days. The follow up period was 4 weeks after the final hijama session [37].
iii. Participants allocated to the intervention groups received three sessions of wet cupping therapy per week for 2 weeks [49].

### Item 4: other components of treatment

#### Item 4a

Details of other interventions administered to the cupping group (e.g., acupuncture, moxibustion, massage, herbs, exercises, lifestyle advice).

##### Explanation

Commonly, cupping is integrative or adjunctive to other treatments, such as cupping and massage in the experiment group or placing a needle inside in the cup (needle cupping) [64]. Any additional components of the cupping intervention, including acupuncture, moxibustion, massage, herbs, exercises, or lifestyle advice, whether carried out by the treatment provider or the patient, should be reported. If corresponding reporting guidelines are available, such as STRICTA for acupuncture [23], STRICTOM for moxibustion [24], CONSORT for CHM formula [22], the details of other interventions should be fully reported according to the relevant checklist. If the trial protocol includes the options of prescribed self-help treatments such as qigong or Taichi exercises and/or lifestyle advice such as dietary changes based on cupping-related diagnostic criteria, then these treatments must be reported. The frequency with which these interventions are given and participants’ compliance with this advice should be described.

##### Examples

i. For the treatment group: Pricking and cupping were applied at Dazhui (DU 14), bilateral Feishu (UB 13) and bilateral Zusanli (ST 36)…… The cupping therapy could last for I0–15 min. The patients were meanwhile prescribed with Qu Feng Tiao Ying (QFTY) decoction, which consisted of Di Huang (raw Radix Rehmanniae) 15 g, Mu Dan Pi (Cortex Moutan) 12 g, Chi Shao (Radix Paeoniae Rubra) 12 g, Jing Jie (Herba Schizonepetae) 10 g, Fang Feng (Radix Saposhnikoviae) 10 g, Xiao Hu Ma (Tenne) 12 g, Ma Huang (raw Herba Ephedrae) 10 g, Gui Zhi (Ramulus Cmnamomi) 12 g, Bai Shao (Radix Paeoniae Alba) 12 g, Huang Qi (Radix Astragali) 15 g, He Shou Wu (Radix Polygoni Multiflori Preparata) 15 g, Ye Jiao Teng (Caulis Polygoni Multiflori) 30 g, and Gan Cao (prepared Radix Glycyrrhizae) 10 g…… The decoction should be taken once a day, 6 days constituting one therapeutic course [65].
ii. Disposable stainless-steel acupuncture needles (0.30 mm × 40 mm, Huatuo Acupuncture, Suzhou, Jiangsu, China) were used. After perpendicular insertion, the patients experienced arrival of Qi [a needling sensation including soreness, numbness, heaviness or distention in the local region of Hegu (LI 4) and Quchi (LI 11)]. The needles were retained for 15 min. Then, a disposable plum-blossom needle (Single-head, Huanqiu Acupuncture, Suzhou, Jiangsu, China) was used to tap the Dazhui (GV 14) area 20 times until a small amount of bleeding occurred, then a cup (5 cm diameter Kangzhu vacuum cup, Beijing, China) was applied and retained for 10 min. At the end of treatment, the Dazhui (GV 14) area was disinfected again and covered with sterile gauze for about 3 h [66].

#### Item 4b

Setting and instruction of treatment to the cupping providers and the participants.

##### Explanation

As cupping is performed on naked and exposed skin, the treatment environment should be comfortably warm and make the patients feel relaxed without any psychological stress. Thus, authors should report the setting(s) where the treatments are performed, such as hospitals, private clinics, or patients’ homes, as well as their specific environment conditions (e.g., temperature, light, etc.). For both cupping providers and cupping recipients, it is also important to provide specific instructions for treatment. Specifically, the instruction to patients usually include the purpose of the trial, inclusion criteria, groupings, and intervention allocation, potential outcomes and adverse effects, if any. The expression of instruction should be standardized. For example, describing a sham cupping control as ‘‘another type of cupping’’ may have a different effect on the outcome than saying it is ‘‘a placebo cupping device, but will experience a similar sensation to real cupping therapy’’ [67].

##### Examples

i. Patients were also asked to contact the trial coordinator if they experienced any adverse events…… All patients further received an instruction sheet in German with a summary of treatment advices, see Additional file 1: Appendix S1 [63].
ii. Before the intervention, an oral explanation was given about the study to eligible persons, informed consent was obtained…… Participants were also informed that they would be called 1 month after cupping and asked about changes in their quality of life…… They were advised to avoid bathing and swimming for 12 h after cupping [68].
iii. This clinical trial was performed at the Beijing University of Chinese Medicine…… Experimental conditions: the room temperature was controlled at about 26 ± 1 °C, relative humidity was maintained at 40% ± 10% throughout the experimental process, and direct sunlight indoors was avoided [69].
iv. Specific procedures: the laboratory temperature was set at 25 °C and relative humidity at 30–60% in a room away from direct sunlight, with the indoor and outdoor environments isolated……the subject was asked to stay awake and remain quiet for 10 min, and the experiment was started when the skin temperature and the ambient temperature were balanced [32].

### Item 5: treatment provider background

Description of treatment provider(s) (qualification or professional affiliation, years in cupping practice, and other relevant experience for professional).

#### Explanation

Characteristics of the professional practitioners providing cupping should be reported, including qualification or affiliation, years in cupping practice, as well as any other experience that may be relevant to the trial results. If applicable, the eligibility criteria for treatment providers should be provided, as these will influence the generalizability of the trial results. Such information is useful for authors of systematic reviews in data analysis, especially since the reporting of these characteristics has always been inadequate [70]. For example, in the design of multicenter trials, it is necessary to report the measures used to standardize treatment procedures between practitioners in different centers [71]. If some potential variations between practitioners are inevitable, it is necessary to report the details of difference, and methods to minimize the effects from practitioners to the outcomes. Besides, if non-specialists or patients themselves provide some part of the treatment, the details of any training in advance, or any other measures used for standardizing the treatment regimens are needed to report.

#### Examples

i. Cupping was performed by expert certified physicians, who regularly performed cupping in the clinical setting [42].
ii. Cupping treatment was conducted by qualified Korean medicine doctors who had over 4 years of practical clinical experience that had been acquired after finishing approximately 10 years of education and training (Appendix) [35].
iii. Acupuncture anesthesia and pricking-bloodletting cupping were performed by a licensed acupuncturist with 5 years of clinical experience having graduated from a University of Chinese Medicine [66].
iv. These participants, and a partner, relative or friend, as appropriate, attended a 1-h practical workshop to learn how to use cupping massage. The clinic regularly runs such sessions to teach patients and their partners cupping massage methods for home use. The workshop was led by an experienced teacher and two assistants and it began with an overview of the history, indications and contraindications of cupping massage, followed by its technique, risks and possible side effects. Cupping massage was then demonstrated by the teacher and a patient volunteer. Patients, and those accompanying them, then practiced the cupping massage technique, with staff members providing feedback and suggestions for improvement as needed. Patients practiced until they felt competent to use the cupping massage technique unaided [63].

### Item 6: control or comparator of cupping

#### Item 6a

Rationale for the choice of control or comparator of cupping.

##### Explanation

The rationale for the choice of control or comparator of cupping (e.g., placebo, active, different cupping regimens, routine care, other CM interventions, no treatment) is necessary to be reported and justified in terms of the research question and the methodology [72]. If cupping-like placebo control (sham cupping device) is used, the author should cite or report the relevant materials that support the use of the selected sham cupping device, such as a previous publication or a pilot study. It is also highly recommended to report whether the co-interventions, including conventional treatments, standard care and complementary and alternative medicine (CAM) interventions (e.g., acupuncture, moxibustion), are provided in the control group and the underlying rationale of selections.

##### Examples

i. The participants assigned to the control group had heating pads warmed by hot water applied to the neck and upper trapezius for 10 min, 3 times per week for 2 weeks. Heating pad therapy is one of the most popular treatment tools for neck pain. It is used alone or concomitant with other physiotherapies for neck pain. The surface temperature of the heating pad was maintained at 55 °C, and it was applied by independent physical therapists. A brochure explaining the relationship between video display terminal (VDT) work and diseases, common treatments for patients with evidence of neck pain, advice on the lifestyle modification when performing VDT work and exercise program details were offered to both treatment groups. During the 7-week participation period, neck and shoulder stretching exercises were performed in the office, and two types of deep cervical flexion exercises (i.e., craniocervical flexion and cervical flexion) were encouraged for home use [35].
ii. Progressive muscle relaxation is a systematic technique used to achieve a deep state of relaxation, developed by Edmund Jacobson. It is widely used by patients with chronic pain conditions; however previous research indicates that relaxation techniques might not be better than usual care for chronic neck pain. In this trial, it was applied to prevent patients from dropping out of the trial due to loss of motivation, because otherwise they would have waited for 12 weeks without any intervention. It was also used as an attention control. Progressive muscle relaxation could also easily be learned and applied at home. Participants in this group attended a 1-h session led by a psychologist experienced in delivering relaxation training…… [63].
iii. Using a sham technique ensured blinding of the patients before receiving general anesthesia…… In the sham group, the cup was remained inactive without negative pressure [36].
iv. A thermal treatment was selected as the control because in Germany, locally applied heat is frequently prescribed for and well accepted by patients with musculoskeletal pain. Patients with connective tissue alterations in the shoulder triangle frequently experience neck pain and commonly apply heat locally to relieve it. Evidence from randomized clinical trials (RCTs) documenting the efficacy of locally applied heat in chronic pain conditions is limited. However, local heat causes vasodilation, increases analgesia, and reduces muscle spasm, all of which would support its use in patients with chronic pain conditions [41].

#### Item 6b

Precise description of the control or comparator. If another form of cupping or cupping-like control is used, provide details as for Items 1 to 3 above.

##### Explanation

The components of the control or comparator should be accurately and comprehensively described. If the control is a cupping-like intervention, such as a form of sham cupping, whether it is a change in the design of a device (e.g., a hole on the surface of the cup) to produce little or no negative pressure or whether adhesive is applied between the cup and skin, should be reported [67]. Additionally, the information of preparation, production, quality control and safety assessment of the sham cupping device should be provided. If usual care or active treatment is the comparator, all the components are recommended to be reported in detail. For cupping trials, it is common to select CAM interventions, such as acupuncture, massage, as the comparator(s). If corresponding reporting guidelines are available, such as STRICTA and STRICTOM [23, 24], the details of those comparators should be fully reported according to the relevant checklist. Where co-interventions are provided to both the experimental and control groups, how patients in different groups are treated should be described in detail. If there is a waitlist or no treatment arm, then the period of waiting or whether the patients will receive further treatment after the study needs to be specified.

##### Examples

i. Sham cupping therapy: the same procedures were applied; however the cupping glasses had been prepared with small holes of < 1 mm diameter to release the negative pressure within seconds. The cups were also fixed by means of elastic tapes. The sham treatment followed the procedure described earlier [43].
ii. The therapeutic success of Cupping Glass Massage (CGM) is compared with that of Acupuncture Therapy (ACU) and tested for non-inferiority…… Performance of acupuncture: The points were selected in the style of the GERAC study which prescribes the acupuncture points partly standardized according to Traditional Chinese Medicine (TCM): mandatory acupuncture points on both sides (Bl23,40,60,Ni3), optional 1–4 Ah-Shi points, supplemented to a total of 6 points by the selection of near points in the lumbar area (Bl24,25,31,54,Gb30) and in the thigh region (Gb31, Bl36,37,Ma31,32) as well as a maximum of 4 points for syndrome therapy (kidney-yang deficiency: plus Du-May-4, kidney-yin deficiency: Ni7 instead of Ni3 on both sides, “Wet Cold”: additional MP9, Bl20 on both sides, “Stagnation of Blood and Qi”: additional Bl17 on both sides) and axis and pathway stimulation points (pain only in the area of the gallbladder tract: Gb34 instead of Bl40 and Gb41 instead of Bl60; axis stimulation: maximum 2 punctures and short manual stimulation; possible points: Bl60, Gb34, Dü3). In total, a minimum of 14 and a maximum of 20 needles were placed for 20 min [44].
iii. Wait List Control Group: treatments in the control group were not regulated; instead, patients were asked to continue their self-directed medical care. The patients were, however, asked to refrain from invasive treatments such as injections or acupuncture. They were asked not to change their treatment regimen during the course of the study. Control patients were offered the same treatment as the intervention group once the trial was concluded [73].
iv. The control group (n = 25) underwent the same exercise training alone.
  Exercise training:
  1. A hollow fist was used to lightly beat the affected upper limb(s) from the back of the hand to the shoulder, then from the palm of the hand to the armpit; this was repeated three times.
  2. The shoulder joint was moved forward and backward for 5 min.
  3. The patient was instructed to raise their hands over their head, and clap their palms together; this was repeated 10 times. Patients were then instructed to spread both upper limbs outward and then back toward to the body; this was repeated three times.
  4. Elbow flexion and chest expansion were performed 20 times.
  5. With the patient facing the wall while standing, the straightened contralateral upper limb was placed as high on the wall as was comfortable, with the palm flat against the wall. The index finger and middle finger of the hand on the affected side were then used alternately to move that arm up the wall until the affected upper limb reached the level of the ipsilateral hand without pain.
    This exercise was performed for 30 s. After recovery, the exercise was continued, and was repeated 10 times per session [74].

## Discussion

Cupping is a traditional therapeutic modality with a series of technical procedures. The reliability of evidence by which the efficacy of this intervention needs to be evaluated and further developed depends on well-designed clinical trials, such as RCTs [75]. Complete, accurate and transparent reporting of intervention details, methodology design and findings are essential. Guidelines can help ensure that trial reporting achieves a minimum standard and that results of trials are verifiable. Therefore, this STRICTOC statement aims to improve the reporting quality of cupping interventions in clinical trials.

The STRICTOC checklist, an extension of intervention item of CONOSRT 2010, includes six items and sixteen sub-items. The items cover the contents for reporting cupping interventions and their control/comparator, as well as the selection rationale. The checklist was developed through extensive consultations and solicitation of comments from the experts of the CONSORT Group, the STRICTA Group, the STRICTOM Group, cupping practitioners and authors of cupping clinical trials. In addition to the checklist, we provide the explanations of each item and list examples of good reporting. The researchers of cupping trials can use this checklist in conjunction with the main CONSORT guideline [16]. The extension to CONSORT for non-pharmacological interventions is also highly relevant to cupping trials [20]. There are other extensions to CONSORT that may be relevant, depending on the trial design, including extensions for cluster trials and non-inferiority trials, and the reporting of abstracts and of harm associated with the interventions (e.g., herbal interventions, Chinese herbal medicine formulas, acupuncture, moxibustion, etc.). All CONSORT guidances and extension documents can be found on the CONSORT website (http://www.consort-statement.org).

For better dissemination of the STRICTOC guideline, we are taking the following specific steps. Firstly, we encourage journals to endorse the STRICTOC and, if applicable, we also recommend journals to implement strategies to improve author adherence to these recommendations. These measures can also help peer reviewers and journal editors in reviewing such trials of cupping. Secondly, because we anticipate benefits from the dissemination by EQUATOR Network (http://www.equator-network.org/), we have registered the STRICTOC guideline in the EQUATOR, and will keep information updated. Thirdly, we will introduce this guideline to relevant clinical practitioners and researchers through health care conferences and workshops. All comments are welcome to improve the STRICTOC and keep it relevant.

Although guideline do help improve the quality of reporting cupping trials, there are some limitations still existing. Firstly, this checklist is used for all types of cupping interventions and thus it could not present all detailed items related to one specific type of cupping. For example, with the development of science and technology, some modern cupping techniques that without using traditional devices and manipulations have emerged. If in this case, the innovation of the cupping technique should be reported in detail. Secondly, in the development of STRICTOC, we did not include a large-scale user-based survey to test the practicality of each item. The value of a guideline ultimately depends on its use. Thus, in future iterations of this checklist, the working group will collect broad feedback from potential users to obtain valuable comments to update it.

## Conclusions

Cupping is a traditional complementary therapy with unique technical procedures. A precise reporting standard is necessary and urged for providing more high-quality clinical evidence on its effectiveness and safety. The items of STRICTOC are specific for reporting interventions in clinical trials of cupping. We hope that it can be further optimized by soliciting comments from the experts of CAM societies and researchers of cupping trials. The STRICTOC guideline will be periodically reappraised and further revised to ensure that it always serves the users of clinical trials of cupping.

## Supplementary information

**Additional file 1: Appendix S1.** STRICTOC working group members (in alphabetical order). **Appendix S2.** Consensus meeting experts (in alphabetical order).

## Data Availability

The data and materials relevant to the STRICTOC checklist are included in this manuscript.

## Abbreviations

STRICTOC: standards for reporting interventions in clinical trials of cupping
CONSORT: Consolidated Standards of Reporting Trials
EQUATOR: Enhancing the Quality and Transparency of Health Research
RCTs: randomized controlled trials
STRICTA: revised standards for reporting interventions in clinical trials of acupuncture
STRICTOM: standards for reporting interventions in clinical trials of moxibustion
CM: Chinese medicine
CHM: Chinese Herbal Medicine
CAM: complementary and alternative medicine
