# Supplementary material for: Standards for reporting interventions in clinical trials of cupping (STRICTOC): extending the CONSORT statement
Source: Chin Med. 2020 Jan 31;15:10. doi: 10.1186/s13020-020-0293-2 (PMC6995163; doi:10.1186/s13020-020-0293-2)
Supplement: Supplementary file 1 — Additional file 1: Appendix S1. STRICTOC working group members (in alphabetical order). Appendix S2. Consensus meeting experts (in alphabetical order). [file 13020_2020_293_MOESM1_ESM.docx]

**Appendix S1:**

**STRICTOC working group members (in alphabetical order):**

Zhao-xiang Bian (Hong Kong, China), Chung Wah Cheng (Hong Kong, China), Bao-yan Liu (Beijing, China), Ai-ping Lyu (Hong Kong, China), You-ping Li (Sichuan, China), Jian-ping Liu (Beijing, China), Myeong Soo Lee (Daejeon, Republic of Korea), Wai Ching Lam (Hong Kong, China), David Moher (Ottawa, Canada), Hong-cai Shang (Beijing, China), Xu-dong Tang (Beijing, China), Ran Tian (Hong Kong, China), Tai-xiang Wu (Sichuan, China), Linda LD Zhong (Hong Kong, China), and Xuan Zhang (Hong Kong, China).

**Appendix S2:**

**Consensus meeting experts (in alphabetical order):**

Yao-long Chen (Lanzhou, China), You-ping Li (Sichuan, China), Jia Liu (Beijing, China), Jian-ping Liu (Beijing, China), Hong-cai Shang (Beijing, China), Tai-xiang Wu (Sichuan, China), Feng-yun Wang (Shanghai, China), Ke-hu Yang (Lanzhou, China), Chen Yao (Beijing, China), Guo-qing Zheng (Wenzhou, China), and Jun-hua Zhang (Tianjin, China).
